# Self-reported and digital-trace measures of computer science students’ self-regulated learning in blended course designs

**DOI:** 10.1007/s10639-023-11698-5

**Published:** 2023-03-24

**Authors:** Feifei Han, Robert A. Ellis

**Affiliations:** 1 Institute for Learning Sciences and Teacher Education, Australian Catholic University, Brisbane, QLD Australia; 2 Office of Pro-Vice-Chancellor (Learning & Teaching), Griffith University, Brisbane, QLD Australia

**Keywords:** Self-reported measure, Digital-trace measure, Self-regulated learning, Blended course designs, Computer science students

## Abstract

This study investigated the extent to which self-report and digital-trace measures of students’ self-regulated learning in blended course designs align with each other amongst 145 first-year computer science students in a blended “computer systems” course. A self-reported Motivated Strategies for Learning Questionnaire was used to measure students’ self-efficacy, intrinsic motivation, test anxiety, and use of self-regulated learning strategies. Frequencies of interactions with six different online learning activities were digital-trace measures of students’ online learning interactions. Students’ course marks were used to represent their academic performance. SPSS 28 was used to analyse the data. A hierarchical cluster analysis using self-reported measures categorized students as better or poorer self-regulated learners; whereas a hierarchical cluster analysis using digital-trace measures clustered students as more active or less active online learners. One-way ANOVAs showed that: 1) better self-regulated learners had higher frequencies of interactions with three out of six online learning activities than poorer self-regulated learners. 2) More active online learners reported higher self-efficacy, higher intrinsic motivation, and more frequent use of positive self-regulated learning strategies, than less active online learners. Furthermore, a cross-tabulation showed significant (*p* < .01) but weak association between student clusters identified by self-reported and digital-trace measures, demonstrating self-reported and digital-trace descriptions of students’ self-regulated learning experiences were consistent to a limited extent. To help poorer self-regulated learners improve their learning experiences in blended course designs, teachers may invite better self-regulated learners to share how they approach learning in class.

## Introduction

The coronavirus pandemic (COVID-19) emergency has required higher education learning and teaching around the world to rapidly respond, in particular, to redeploy even more learning and teaching activities to virtual learning spaces to promote physical distancing. As a result, the vast numbers of face-to-face courses have been delivered either as blended courses or as purely online courses (Tang et al., 2021). Learning in the online and blended contexts requires students to take high levels of control of and to regulate their learning (Vanslambrouck et al., 2019; Wong et al., 2019). As a result, an increasing number of studies have examined students’ self-regulated learning in online and blended deliveries (Broadbent & Fuller-Tyszkiewicz, 2018; Sun et al., 2018). These studies have focused on how different aspects in self-regulated learning, such as motivation and emotion, may impact on students’ learning processes and their learning outcomes (Li et al., 2020). However, these studies have predominantly employed self-reported measures to examine what and how students learn in online and blended contexts (Han, 2022). With the development of modern technology and learning analytics, the online learning management systems (LMSs) are able to record students’ online learning and generate digital-trace data to describe what and how students learn online (Ainley & Patrick, 2006). In this scenario, it is important to examine the extent to which self-reported and digital-trace measures are consistent with each other in terms of describing students’ self-regulated learning, which is the focus of this chapter.

## Literature review

### Social-cognitive view of self-regulated learning

Self-regulated learning describes learners’ cognitively and metacognitively oriented thoughts, feelings, and actions towards the attainment of certain learning goals (Zimmerman & Schunk, 2001). It recognizes the active role played by students and acknowledges the self-directed nature in the process of learning, as students are thought to be able to set goals for their learning; monitor, regulated, and modify their cognition and motivation in order to achieve the learning goals they set (Zimmerman, 2000). During the constant monitoring, regulating, and modifying processes, learning takes place by students’ active construction of meaning through an interaction between their prior knowledge, their own characteristics, and their learning contexts and environments (Pintrich, 2000). Various models have been proposed to describe and conceptualize self-regulated learning (Moos & Stewart, 2013). Some models conceive self-regulated learning as an event-based phenomenon in specific contexts (Azevedo, 2009; Azevedo et al., 2010; Greene & Azevedo, 2010; Järvelä & Hadwin, 2013); whereas others describe the overall process of self-regulated learning (Zimmerman, 2000). The self-regulated models also have different theoretical bases. For instance, Winne and Hadwin’s (1998) model adopts an information processing perspective; the model proposed by McCaslin and Hickey (2001) departs from a sociocultural point of view; and Pintrich’s (2000) self-regulated model is based on social-cognitive theory. Researchers have also attached importance to different aspects in the process of self-regulated learning. While some researchers emphasize metacognitive monitoring and control in self-regulated learning (Winne, 2001; Winne & Perry, 2000); some focus on goal settings (Boekaerts, 2011); and others pay attention to the cognitive aspect of self-regulated learning (Winne, 1996).

Amongst different conceptualised models of self-regulated learning, Pintrich’s (2000) social-cognitive model comprehensively describe the main process of self-regulated learning and is one of the most widely adopted theoretical framework to measure self-regulated learning (Broadbent & Poon, 2015). This model proposes a triadic reciprocal interaction amongst motivation, the environment, and behaviour, in which self-regulated learning is perceived as a “dynamic and contextually bound” phenomenon rather than a static trait of students (Duncan & McKeachie, 2005, p. 117). It proposes that learners’ motivation, cognition, and self-regulated learning strategies may vary depending on the contextual features of the learning environment and characteristics of learning tasks (e.g., the nature of the course, its structure, and the learning activities) along with learners’ internal state of mind (e.g., students’ interest and their abilities) (Pintrich & Zusho, 2007). Therefore, the external environment, such as the course designs (including the design of the online course sites) can either facilitate or hamper self-regulated learning processes (Zimmerman & Schunk, 2011), and may in turn affect the academic performance (Broadbent & Poon, 2015). In this perspective, able self-regulated learners are often described as having higher self-efficacy to manage and effectively organize study with a minimum of distractions, feeling less anxious, having appropriate learning goals, and adopting appropriate self-regulated learning strategies (Pajares, 2002). Based on this model, Pintrich and colleagues developed the Motivated Strategies for Learning Questionnaire (MSLQ, Pintrich et al., 1991), which is one of the most frequently adopted instrument to measure self-regulated learning (Azevedo et al., 2012).

### Research on self-regulated learning using self-reported measures

According to Winne and Perry (2000), self-regulated learning has properties of an aptitude and an event. The aptitude aspect concerns relatively stable attributes of a person which influence his/her behaviors; whereas the event aspect describes self-regulated learning as occurrences marked by transitions from one state to another along a timeline. Investigations into the aptitude aspect of self-regulated learning have predominantly relied on collecting self-reported data, such as questionnaires, structured interviews, teacher judgement, or think-aloud method (Schellings & Van Hout-Wolters, 2011; Weinstein et al., 1987; Winne, 2010). Of these methods, self-reported questionnaires are the most frequently used measures due to the easiness of administer and score (Winne & Perry). A considerable number of aptitude variables in self-regulated learning have been researched, such as motivation, anxiety, concentration, time management, and strategies (Panadero, 2017).

However, the self-reported data have received criticism, such as lacking objectivity (Matcha et al., 2020; Zhou & Winne, 2012); students’ careless answering and item nonresponse (Hitt et al., 2016; Zamarro et al., 2018); their inability to represent the complexity and dynamics of students’ self-regulated strategy use in real learning contexts (Zhou & Winne, 2012). To improve insights into understanding students’ self-regulated learning, researchers have suggested to use multiple measures and types of data, such as digital-trace data (Vermunt & Donche, 2017), as this type of data may provide “both researchers and practitioners with the opportunity to monitor students’ strategic decisions in online environments in minute detail and in real time” (Richardson, 2017, p. 359).

### Research on self-regulated learning using digital-trace measures

The rich and detailed digital-trace of students’ interactions with a variety of online learning resources and activities have the advantage of offering descriptions of students’ online learning behaviors and strategies relatively more objectively than using self-reported measures (Baker & Siemens, 2014; Siemens, 2013; Sclater et al., 2016).

For instance, Jovanović et al. (2017) used the 13-week digital-trace data extracted from the LMS to investigate 290 computer science students’ self-regulated online learning strategies. The hierarchical sequence analysis identified five distinct of groups of students differed by their self-regulated learning strategies, namely *the intensive students* (diverse online learning strategies); *the strategic students* (focusing on summative and formative assessments); *the highly strategic students* (focusing on summative assessments and reading materials); *the selective group* (emphasizing summative assessments without much involvement in reading the course materials); and *the highly selective group* (predominantly performing summative assessments). The students with different self-regulated learning strategies also differed on their academic performance: the first three groups performed significantly better on both mid-term and final examinations than the other two groups. Despite presenting informative findings with regard to students’ use of self-regulated learning strategies in online learning, this study failed to reveal information on learners’ internal state of mind, such as their motivation, learning goals, and anxiety, which are important aspects in social-cognitive perspectives on self-regulated learning.

### Research on self-regulated learning by combining self-reported and digital-trace measures

Recognising the limitations of the self-reported and digital-trace measures to research self-regulated learning, researchers have proposed to combine both self-reported and digital-trace measures, which will enable investigations of students’ self-regulated learning to be more holistic and comprehensive (Gašević et al., 2015; Lockyer et al., 2013; Rienties & Toetenel, 2016). The majority of the existing studies combining self-reported and digital-trace measures examined how combining two types of measures would improve the explanatory power to predict the academic performance of self-regulated learning (Reimann et al., 2014). For instance, using multiple regression analyses, Pardo et al. (2017) found that students’ reported anxiety in learning and their reported use of self-regulated learning strategies could only explain 7% of variance in students’ academic performance; whereas adding the frequency of students’ interactions with the online learning activities measured by digital traces into the regression model could explain 32% of variance in students’ academic performance, which increased an additional of 25% of variance. Little research, however, investigates the extent to which the two types of measures are consistent to describe students’ self-regulated learning, which will be the aim of the current study.

The current study sought to answer three research questions:

1. Do digital-trace measures of students’ interactions with the online learning activities and academic performance differ by their self-reported self-regulated learning?
2. Do self-regulated learning reported by students and academic performance differ by digital-trace measures of students’ interactions with the online learning activities?
3. Do profiles of students’ self-regulated learning by self-reports and digital traces align with each other?

## Method

### Participants and the context of research

The participants were 145 computer science undergraduates, of whom 108 were male (74.5%) and 37 were female (25.5%). They were enrolled in a first-year compulsory course – “computer systems” in an Australia metropolitan university. The course run for 13 weeks and had four learning aims: 1) to understand the concepts of computer systems; 2) to gain insights into fundamentals of information technology hardware and software; 3) to distinguish different types of business systems and to conduct basic systems analysis; and 4) to study software development and create simple Java programs. In addition to acquiring content knowledge and skills, the course also aimed to horn students’ generic attributes, such as independent inquiry skills, communication and negotiation abilities; and developing information and digital literacy. The course was designed as a blended course, which not only required students to attend face-to-face and online learning and teaching but also to study online learning materials and activities in a customarily designed LMS (Please see the instrument section for online learning activities).

### Instruments

Three types of data were collected, namely self-reported data, digital-trace data, and academic performance data.

#### Self-reported data collected by the motivated strategies for learning questionnaire (MSLQ)

The self-reported data were collected using five scales in the MSLQ (Pintrich et al., 1991), namely self-efficacy (8 items), anxiety (4 items), intrinsic motivation (5 items), positive self-regulated learning strategies (4 items), and negative self-regulated learning strategies (3 items). The MSLQ was developed and validated by Pintrich and colleagues (Pintrich et al.). The questionnaire asked students to rate on 7-point anchors, with 1 representing “not at all true of me” and 7 indicating “very true of me”. In our study, the values of Cronbach’s alpha reliability of all the five scales (self-efficacy: α = .89, anxiety: α = .84, intrinsic motivation: α = .85, positive self-regulated learning strategies: α = .68, and negative self-regulated learning strategies: α = .68) were above .65, which showed acceptable reliability (Griethuijsen et al., 2014).

#### Digital-trace data of students’ interactions with the online learning activities

The digital-trace data were collected via the learning analytics function built in a customer-designed LMS, which was able to record frequency of students’ interactions with the online learning activities. There were six types of online learning activities:

- dashboard-view: provided a student’s performance with regard to other students in the course.
- collapse-and-expand: involved collapsing and expanding HTML page.
- resource-view: involved viewing the supplementary course materials.
- video: required learners to watch a video file.
- multiple-choice-question: required learners to complete a multiple-choice-question.
- excise: required students to answer questions in a sequence.

#### Academic performance

Students’ course marks were used to represent their academic performance. The course mark was an aggregated score of the six assessment tasks, with the highest possible mark being 100. The six assessment tasks were: 1) weekly online exercises (10%); 2) tutorial preparation and participation (10%); 3) written laboratory reports, which included laboratory experiment logs, and problems and solutions of laboratory activities and work (5%); 4) major project (15%); 5) midterm examination (20%); and 6) final examination (40%).

### Data collection procedure

Data collection strictly followed the ethical procedures stipulated in the Human Ethics Committee of the researcher’s University. Before the data collection, we explained to the participants that participating in the study was completely voluntary and obtained their written consent to completing the questionnaire, to access to the digital footprints left in the LMS, and to access to their course marks. We explained to the participants that the data would only be used for research purposes and their identity would be kept anonymously throughout the research. We administered the questionnaire towards the end of the course, which allowed students to have relatively full experience to reflect upon.

### Data analysis

Methods for data analyses are presented in Table 1.

**Table 1:** Methods for data analyses

Research questions	Methods
1. Do digital-trace measures of students’ interactions with the online learning activities and academic performance differ by their self-reported self-regulated learning?	1a) a hierarchical cluster analysis using self-reported measures to cluster students; 1b) one-way ANOVAs using digital-trace measures as the dependent variables and the cluster membership generated in 1a) as the independent variable
2. Do self-regulated learning reported by students and academic performance differ by digital-trace measures of students’ interactions with the online learning activities?	2a) a hierarchical cluster analysis using digital-trace measures to cluster students; 2b) one-way ANOVAs using self-reported measures as the dependent variables and the cluster membership produced in 2a) as the independent variable
3. Do profiles of students’ self-regulated learning by self-reports and digital traces align with each other?	a cross-tabulation using students’ clusters resulted from the above mentioned two hierarchical cluster analyses

## Results

### Descriptive statistics

The descriptive statistics of self-reported measures, digital-trace measures, and academic performance are presented in Table 2.

**Table 2:** Descriptive statistics

Variables	Minimum	Maximum	*M*	*SD*
Self-reported measures				
Self-efficacy	2.00	7.00	4.75	1.00
Anxiety	1.00	7.00	3.62	1.36
Intrinsic motivation	2.80	7.00	5.53	0.96
Positive self-regulated learning strategies	2.25	7.00	4.55	1.05
Negative self-regulated learning strategies	1.00	7.00	3.41	1.19
Digital-trace measures				
Dashboard-view	0.00	233.00	31.10	41.84
Collapse-and-expand	59.00	1182.00	421.97	234.36
Resource-view	138.00	2492.00	818.07	443.00
Video	10.00	2890.00	338.59	395.48
Multiple-choice-question	10.00	3054.00	233.01	300.50
Exercise	353.00	9957.00	2723.49	1419.81
Academic performance				
Course marks	20.00	98.50	65.55	16.12

### Results for research question 1 – Differences of digital-trace measures of students’ online learning and academic performance based on self-reported measures

The increasing value of the squared Euclidean distance between the clusters suggested a two-cluster solution: cluster 1 had 62 students whereas cluster 2 had 83 students (Table 3). One-way ANOVAs showed significant differences on all the self-reported measures: self-efficacy, anxiety, and intrinsic motivation, positive self-regulated learning strategies, and negative self-regulated learning strategies. Specifically, cluster 1 students reported higher self-efficacy, intrinsic motivation, and use of positive self-regulated learning strategies; but lower anxiety, and use of negative self-regulated learning strategies, than cluster 2 students. According to these patterns, cluster 1 students were named as better self-regulated learners and cluster 2 students were called poorer self-regulated learners.

**Table 3:** The results of the one-way ANOVAs based on self-reported measures

Variables	Better self-regulated learners (*n* = 62)	Poorer self-regulated learners (*n* = 83)	*F*	*p*	η^2^
	*M*	*M*			
Self-reported measures					
Self-efficacy	5.30	4.35	41.15	**.00**	.22
Anxiety	3.24	3.90	8.74	**.00**	.06
Intrinsic motivation	5.98	5.18	29.72	**.00**	.17
Positive self-regulated learning strategies	5.23	4.05	63.46	**.00**	.31
Negative self-regulated learning strategies	2.69	3.95	55.00	**.00**	.28
Digital-trace measures					
Dashboard-view	41.84	23.07	7.46	**.01**	.05
Collapse-and-expand	439.65	408.76	0.62	.43	.00
Resource-view	879.63	772.08	2.11	.15	.02
Video	387.23	302.25	1.65	.20	.01
Multiple-choice-question	291.18	189.57	4.15	**.04**	.03
Exercise	3036.02	2490.04	5.41	**.02**	.04
Academic performance					
Course marks	68.81	63.11	4.54	**.04**	.03

Furthermore, one-way ANOVAs also showed that better and poorer self-regulated learners differed significantly on the frequencies of the interactions of the three out of six online learning activities, namely dashboard-view; multiple-choice-question; and exercise. Better self-regulated learners interacted significantly more frequently than their peers in the poorer self-regulated cluster on dashboard-view, multiple-choice-question, and exercise. Moreover, better self-regulated learners also achieved significantly better academic performance than poorer self-regulated learners.

### Results for research question 2 - differences of self-reported measures of students’ self-regulated learning and academic performance based on digital-trace measures

Based on the increasing value of the squared Euclidean distance between the clusters, the hierarchical cluster analysis using digital-trace measures also produced a two-cluster solution: cluster 1 and 2 had 88 and 57 students respectively (Table 4). One-way ANOVAs found significant differences between the two clusters on all digital-trace measures: dashboard-view, collapse-and-expand view, resource, video, multiple-choice-question, and exercise. Specifically, cluster 1 students had higher frequencies of the interactions with all the online learning activities than their peers in cluster 2. Hence, cluster 1 and cluster 2 were referred to as more active and less active online learners respectively.

**Table 4:** The results of the one-way ANOVAs based on digital-trace measures

Variables	More active online learners (*n* = 88) *M*	Less active online learners (*n* = 57) *M*	*F*	*p*	η^2^
Digital-trace measures					
Dashboard-view	44.56	10.32	27.42	**.00**	.16
Collapse-and-expand	536.25	245.53	83.86	**.00**	.37
Resource-view	1040.89	474.07	92.69	**.00**	.39
Video	484.81	112.84	38.59	**.00**	.21
Multiple-choice-question	321.23	96.82	22.12	**.00**	.13
Exercise	3494.40	1533.32	120.99	**.00**	.46
Self-reported measures					
Self-efficacy	4.90	4.53	4.80	**.03**	.03
Anxiety	3.62	3.63	0.01	.94	.00
Intrinsic motivation	5.67	5.30	5.45	**.03**	.04
Positive self-regulated learning strategies	4.79	4.18	12.31	**.00**	.08
Negative self-regulated learning strategies	3.39	3.45	0.10	.75	.00
Academic performance					
Course marks	70.18	58.39	21.12	**.00**	.13

The one-way ANOVAs further revealed that more active and less active online learners also differed significantly on three out of five self-reported scales: self-efficacy, intrinsic motivation, and positive self-regulated learning strategies. More active online learners reported having higher self-efficacy, higher intrinsic motivation, and using more positive self-regulated learning strategies, than less active online learners. At the same time, the two clusters of students also differed significantly on their final examination results. More active online learners obtained higher course marks than less active online learners.

### Results for research question 3 - alignment of profiles students by self-reported and digital-trace measures

The results of the cross-tabulation analysis revealed significant but weak association between the clusters by self-reported and digital-trace measures: χ^2^ (1) = 8.28, *p < .*01, φ = .24. Table 5 shows that amongst students who self-reported as better self-regulated learners, a significantly higher proportion was more active online learners (74.2%) than less active learners (25.8%). However, of students who self-reported as poorer self-regulated learners, the proportions between more (50.6%) and less (49.4%) active online learners did not differ.

**Table 5:** Results of the cross-tabulation analysis

Self-reported clusters	Count % within digital-trace clusters	More active online learners	Less active online learners	total
Better self-regulated Learners	count	46_a_	16_b_	62
	% within digital-trace clusters	74.2%	25.8%	100.0%
Poorer self-regulated learners	count	42_a_	41_a_	83
	% within digital-trace clusters	50.6%	49.4%	100.0%
total	count	88	57	145
	% within digital-trace clusters	60.7%	39.3%	100.0%

Different subscript letters denote a subset of self-reported clusters whose column proportions differ significantly from each other at the .05 level

## Discussion

### Clustering students based on self-reported or digital-trace measures

We found a certain level consistency no matter using self-reported or digital-trace measures to cluster students. The cluster analyses identified groups of students who showed similar self-regulated learning experience in terms of how they reported their efficacy, anxious feelings, intrinsic goals, and their self-regulated learning strategies on the one hand, and how they were observed to engage online, and how well they achieved in the course. Within each group, there was coherence between self-reported learning experience and their actual online engagement in the compulsory online part of the course. Students who self-reported having higher efficacy, setting higher intrinsic goals, feeling less anxious, using more appropriate self-regulated learning strategies in the course, were also more likely to be active learners in the online part of the learning, as they tended to use the dashboard functions to evaluate their learning by comparing it with other students; interacted with the multiple-choice-questions and exercises significantly more frequently than those who reported lower efficacy, lower intrinsic goals, more anxiety, and using less desirable self-regulated learning strategies. At the same time, better self-regulated learners measured by self-reporting also scored significantly higher in the course performance than poorer self-regulated learners did. Oure results were consistent with previous studies, which reported that self-regulated learners also tended to achieve better academic performance (Kizilcec et al., 2013, 2017).

### Weak association between students’ profiles by self-reported and digital-trace measures

Furthermore, although we found significant association (*p* < .01) between students’ profiles by self-reported measures and digital-trace measures, such association was rather weak (φ = .24). Of students who were classified as poorer self-regulated learners by their reporting on their self-efficacy, intrinsic goals, anxiety, and use of regulatory strategies in learning, the proportions of more active and less active online learners by digital-trace measures were the same (50.6% vs. 49.4%). One possible explanation of this finding could be that poorer self-regulated learners were less accurate in their self-reporting. This weak alignment was also reported in Ye and Pennisi (2022) that approximately one third of their participants (from 30.8% to 53.9% depending on whether there were three or two types of self-regulated learning strategies) demonstrated inconsistency between their self-regulated learning strategies measured by self-reported questionnaires and digital-trace data. Ye and Pennisi also found that amongst their participants, better self-regulated learners tended to report their self-regulated learning experience more accurate than poorer self-regulated learners. Indeed, in the interviews with the participants, Ye and Pennisi found that better self-regulated learners were able to identify their weaknesses through self-reflection – an important self-regulatory ability.

Another possible explanation of weak alignment between students’ profiles by self-reported and digital-trace measures could be that the two types of measures captured different aspects in self-regulated learning and could offer complementary information. Past research demonstrated that inclusion of both students’ self-regulated learning experience assessed by self-reported measures (e.g., use of self-regulated learning strategies) and digital-trace measures (e.g., frequency of students’ online learning interactions) significantly increased the percentage of predictions of academic achievement than using either self-reported measures or digital-trace measures alone. For example, Pardo et al. (2017) found that students’ reporting of self-regulated learning predicted only 7% of variance in their academic achievement. Adding digital traces of online interactions predicted an additional of 25% of variance in students’ academic achievement. Similarly, Li et al. (2020) found that adding digital-trace measures of students’ time management (regulatory learning strategies) on top of students’ self-reporting about how they managed time during the course improved the prediction from 1% to 30% for course grade, and from 1% to 28% for final examination score. In the same study, Li et al. also showed adding digital traces of students’ effort regulation into the regression analyses increased the predictive power made by just using students’ reporting of their effort regulation from 2% to 28% for course grade, and from 2% to 24% for final examination scores. Viewed together, these past findings as well as ours may suggest that combining using self-reporting and digital traces measures is likely to offer a more comprehensive picture of students’ self-regulated learning experience in blended course designs than using either measure alone.

### Implications of the study

The results of the study may not only benefit teachers and students but also institutional learning designers. The identification of the better self-regulated learners early in the course may allow teachers to invite those learners to share their experience, such as how they direct their actions and efforts to achieve their learning goals, so that poorer self-regulated learners can make adjustment to emulate their peers. To encourage students to actively participate in the online learning, teachers may consider adopting strategies which aim to orient students to online learning and to the online learning site. For example, teachers may explicitly explain the purposes of different online activities, resources, and materials, and how these are linked with the learning and teaching in the face-to-face lectures. Teachers may also guide students to navigate the online course site and to explore various functions and sections in the LMS.

As our study also showed that digital-trace measures extracted from LMS were able to reflect how students approach online learning activities and tasks differently, it will benefit students if learning designers can improve functions of LMS to embed learning analytic tools which allow students to monitor their own online learning, as currently most learning analytic tools are designed solely for the teaching staff (Viberg et al., 2018). For instance, tools can be built to alert students if their online participation is below the class average. This will remind students with low online participation to catch up.

### Limitations and directions for future research

Some of the limitations of the study should be taken into consideration when interpreting the results and designing further research on this line of inquiry. First, the major limitation of the study was that using frequency of interactions with the online learning activities alone to represent students’ online learning interaction. Frequency of online participation is only one of the possible indicators of students’ online learning. Apart from frequency, duration of time spent on different online learning activities and patterns of time-stamped sequences of online learning activities are also possible indicators of students’ online learning (Bannert et al., 2014; Hadwin et al., 2007; Han et al., 2022; Winne et al., 2017). Therefore, future studies should use different types of digital-trace data to assess students’ online learning (Fincham et al., 2019; Jovanović et al., 2017).

Second, digital-trace measures of students’ interactions with the online learning activities only reflected students’ learning experience in the online part of the whole course, whereas self-reported measures of students’ self-efficacy, intrinsic goal, anxiety, and self-regulated learning strategies were concerned with the learning in both face-to-face and online components in the course. Future research should add additional more objective measures (other than self-reported measures) of students’ learning experiences in the face-to-face part of the learning in blended course designs.

## Data Availability

The dataset used and analysed during the current study is available from the corresponding author on reasonable request.
